# Microstructure Evolution and Strength–Ductility Synergy of Diffusion-Bonded Rare-Earth Magnesium Alloy Joints Under Electric Current Treatment

**DOI:** 10.3390/ma19194229

**Published:** 2026-10-05

**Authors:** Feng He, Chong Du, Zhichao Jia, Yanli Song, Ye Li, Jue Lu, Yanxiong Liu

**Affiliations:** 1 State Key Laboratory of Light Superalloys, Wuhan University of Technology, Wuhan 430070, China; 2 Hunan Vanguard Group Co., Ltd., Changsha 410082, China; 3 Hubei Key Laboratory of Advanced Technology for Automotive Components, Wuhan University of Technology, Wuhan 430070, China

**Keywords:** Mg-Gd-Y-Zn-Zr rare-earth magnesium alloy, diffusion bonding, electroshock treatment, diffusion interface, mechanical properties

## Abstract

Diffusion bonding provides an effective approach for joining rare-earth magnesium alloys; however, achieving a desirable combination of interfacial integrity and ductility in the bonded joints remains challenging. In this work, an electroshock treatment (EST) process has been proposed to improve the microstructure and mechanical properties of diffusion-bonded joints of Mg-Gd-Y-Zn-Zr rare-earth magnesium alloys. Diffusion bonding of the rare-earth magnesium alloy was first performed using an SPS apparatus, followed by EST of the diffusion-bonded joint under varied processing parameters. The experimental results demonstrate that at an EST current density of 50 A/mm^2^ and a treatment duration of 15 s, the elongation at fracture of the diffusion-bonded joint increases from 2.42% to 3.60%, corresponding to an enhancement of 48.8%, while only a limited change in the ultimate tensile strength is observed. Microstructural characterization indicates local Y enrichment at the diffusion bonding interface. These changes may be associated with the combined influence of Joule heating and possible athermal effects of the applied current; however, their individual contributions cannot be distinguished in the present study. While the electron backscatter diffraction (EBSD) results suggest a redistribution of geometrically necessary dislocations (GNDs) and a tendency toward texture weakening within the analyzed regions. These microstructural changes contribute to the enhanced ductility of the diffusion-bonded joint after EST.

## 1. Introduction

With the growing demand for lightweight materials in the aerospace, consumer electronics, and automotive industries, magnesium and its alloys have attracted considerable attention for their low density, high specific strength, excellent damping capacity, superior electromagnetic shielding performance, and recyclability [1,2,3,4]. However, compared with other lightweight alloys such as aluminum and titanium alloys, conventional magnesium alloys suffer from low high-temperature strength and poor ductility, which limits their application in critical load-bearing components. At present, alloying modification (e.g., the addition of rare-earth elements) is an effective means of enhancing the comprehensive mechanical properties of magnesium alloys [5,6,7]. Relevant studies have demonstrated that rare-earth elements can achieve synergistic regulation of the microstructure and mechanical performance of magnesium alloys by promoting dynamic recrystallization, activating non-basal slip systems, and inducing the formation of a rare-earth texture [8,9,10]. Rare-earth-modified magnesium alloys exhibit remarkable improvements in heat resistance, corrosion resistance, strength, and toughness, as well as high-temperature creep resistance, thus presenting superior overall service performance [11,12,13].

Although the performance improvement of rare-earth magnesium alloys provides fundamental support for their engineering applications, their reliable joining remains a critical bottleneck that restricts the fabrication and service of structural components. At present, the mainstream welding processes for magnesium alloys include fusion welding [14], friction stir welding [15], and brazing [16]. During fusion welding of magnesium alloys, severe oxidation often occurs, leading to the formation of refractory oxide inclusions. Furthermore, the volatilization of alloying elements and the escape of gases from the molten pool readily induce pores and other defects, which degrade the mechanical properties of the welded joints. In contrast, although friction stir welding can effectively avoid the defects inherent to diffusion bonding, it is constrained by welding path planning and the geometric configuration of components. As a result, it is only applicable to the joining of regular and simple structures and cannot satisfy the integral welding requirements of complex components.

As a typical solid-state joining technology, diffusion bonding connects metal components through local plastic deformation and interfacial atomic diffusion under specific temperature and pressure conditions [17,18]. It can restrain welding deformation to the greatest extent due to the absence of melting and rapid cooling. Diffusion bonding operates at a lower process temperature and avoids the formation of a heat-affected zone, thereby effectively preserving the intrinsic mechanical properties of the magnesium alloy base material [19]. Therefore, it provides an ideal technical approach to overcome the thermal damage issues in magnesium alloy welding [20]. Tong et al. [21] conducted vacuum diffusion bonding experiments on as-cast Mg-Gd binary alloys at different temperatures. The results showed that the maximum tensile strength of the joint reached 151 MPa at a bonding temperature of 550 °C, and the elongation after fracture was 119.1% of that of the as-cast base metal. Within the low-temperature eutectic range, Gd atoms diffuse toward the welding interface and form Gd-rich precipitates, thereby enhancing interfacial bonding strength. Ma et al. [22] investigated the diffusion bonding and post-weld heat treatment of Mg-6Gd-3Y rare-earth magnesium alloy. Their results indicate that rare-earth elements and their intermetallic compounds tend to segregate and accumulate at the bonding interface, which remarkably inhibits grain boundary migration across the interface. After the synergistic application of diffusion bonding at 440 °C and multi-stage heat treatment, the ultimate tensile strength of the joint reaches 279 MPa, with an elongation of 2.8%.

Electroshock treatment (EST) has gradually attracted extensive attention from researchers due to its unique advantages in performance enhancement [23,24]. By applying an instantaneous high-energy current to the atoms within a material, EST promotes the ordered rearrangement of atoms in high-energy metastable regions and homogenizes the internal stress distribution. This innovative technology utilizes transient alternating energy to regulate microstructural defects in metallic materials. Wu et al. [25] applied EST to a 5% pre-deformed Al–Zn–Mg–Cu alloy to improve its ductility. Microstructural observations reveal that EST promotes dislocation homogenization and the formation of dislocation forests, leading to a 37.9% increase in elongation relative to that of the untreated alloy. Chen et al. [26] employed EST on M50 bearing steel, achieving respective improvements of approximately 7.02% in elongation at break and 26.91% in impact toughness. Microstructural analysis reveals that EST promotes a homogeneous distribution of geometrically necessary dislocations (GNDs) while accelerating the migration and annihilation of high-energy dislocations, thereby enhancing the overall mechanical properties of the steel. In addition, EST has exhibited significant performance improvement effects on various other materials, including titanium alloys, aluminum alloys, and steels [27,28,29]. These findings indicate that EST is applicable to the performance enhancement of most engineering alloys.

In contrast, spark plasma sintering (SPS), developed based on SPS technology, has rapidly emerged in recent years with benefits such as low-temperature operation, minimal deformation, and high efficiency. It enables material joining under combined electrical, thermal, and pressure loading [30]. Therefore, the present study applies spark plasma sintering (SPS) diffusion bonding to a Mg-Gd-Y-Zn-Zr rare-earth magnesium alloy. The resultant bonded joints are subsequently subjected to EST to evaluate the influence of this process on their mechanical properties. Scanning electron microscopy (SEM) and electron backscatter diffraction (EBSD) characterization are performed before and after diffusion bonding and before and after EST to analyze microstructural evolution. This work aims to clarify the influence of post-bonding EST on the microstructural evolution of Mg-Gd-Y-Zn-Zr rare-earth magnesium alloy joints and further reveal the underlying mechanism for the improvement in elongation of diffusion-bonded joints induced by EST.

## 2. Materials and Methods

### 2.1. Microstructure and Mechanical Properties of Rare-Earth Magnesium Alloy

In this study, multi-directional forged (MDF) Mg-Gd-Y-Zn-Zr rare-earth magnesium alloy plates, 60 mm thick, were selected as the experimental base material. The actual chemical composition of the alloy was measured using an inductively coupled plasma optical emission spectrometer (ICP-OES; Agilent Technologies model 730, Santa Clara, CA, USA), and the measured mass fractions of each element are listed in Table 1.

**Figure 1:**
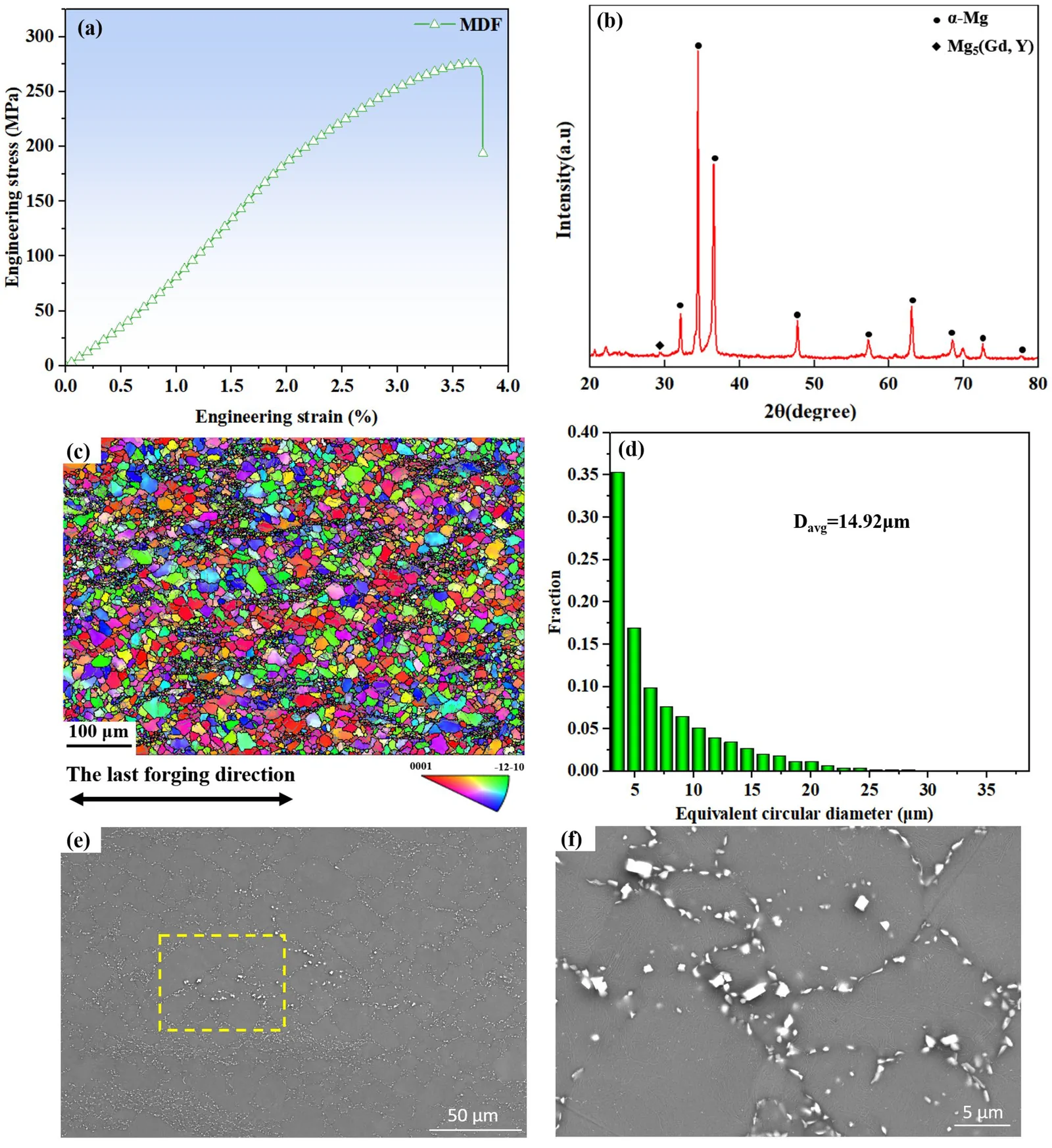
Mechanical properties and microstructure of the initial rare-earth magnesium alloy: (**a**) tensile curves, (**b**) XRD pattern, (**c**) IPF map, (**d**) grain size statistics, and (**e**) SEM microstructure, and (**f**) enlarged SEM image of the region marked by the yellow dashed box in (**e**).

The microstructural characteristics and mechanical properties of the as-received multi-directionally forged Mg-Gd-Y-Zn-Zr rare-earth magnesium alloy plate are presented in Figure 1a–d. As shown in Figure 1a, the as-received plate exhibits an ultimate tensile strength of 285 MPa and an elongation after fracture of 3.75%. The phase composition analyzed via X-ray diffraction (XRD) is shown in Figure 1b. The MDF Mg-Gd-Y-Zn-Zr alloy is predominantly composed of the α-Mg matrix, along with a minor fraction of the face-centered-cubic (FCC) Mg_5_(Gd,Y) phase [31]. The inverse pole figure (IPF) maps and grain size distribution diagram of the MDF magnesium alloy are shown in Figure 1c,d. The alloy matrix exhibits a mixed-grain microstructure characterized by the coexistence of dynamically recrystallized grains and deformed grains. Owing to plastic deformation during multi-directional forging, the initial, as-cast grains are significantly refined and fragmented. A large number of fine equiaxed recrystallized grains are preferentially distributed along the deformed grain boundaries, resulting in an overall grain refinement with a gradation in grain size distribution. The large grains exhibit a high internal misorientation, whereas the fine dynamically recrystallized grains possess a relatively uniform orientation. The average grain size of the MDF microstructure was measured to be 14.92 μm. The SEM microstructure of the initial MDF alloy is shown in Figure 1e,f, in which bright second-phase particles are distributed within the Mg matrix.

### 2.2. Diffusion Bonding

**Figure 2:**
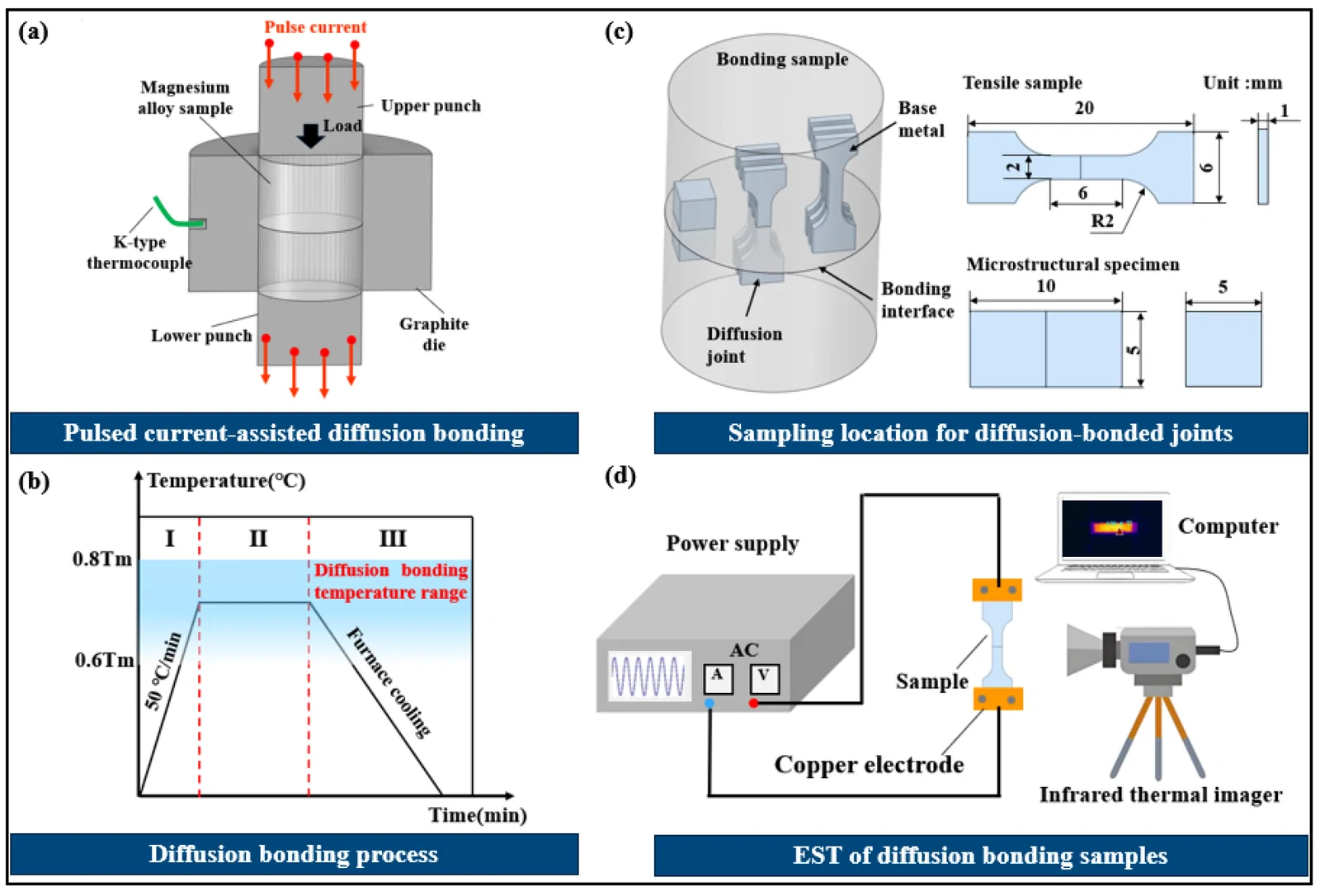
Schematic diagram of diffusion bonding specimen and electroshock treatment: (**a**) pulsed current-assisted diffusion bonding, (**b**) diffusion bonding process, (**c**) sampling locations for diffusion-bonded joints, (**d**) EST of the diffusion-bonded samples.

Cylindrical specimens with a diameter of 30 mm and a thickness of 20 mm were cut via wire electrical discharge machining (WEDM). The bonding end faces of the specimens were sequentially ground and polished using abrasive papers from #600 to #2000 grit. Subsequently, the specimens were ultrasonically cleaned in acetone solution to remove surface oil contamination and impurities, followed by drying with cold air to prevent secondary surface contamination. The pretreated and dried specimens were assembled in an SPS vacuum diffusion furnace (SINTER LAND INC model LABOX-1575, Nagaoka, Japan) prior to welding, and the specimen placement is illustrated in Figure 2a. To effectively suppress high-temperature oxidation at the welding interface and ensure high interfacial bonding quality, the solid-state diffusion bonding experiment was conducted while maintaining the furnace vacuum level below 5 Pa throughout the entire process. As shown in Figure 2b, the process curve of diffusion bonding comprises three stages: pressure loading and heating, isothermal diffusion, and furnace cooling. In Stage I, a constant bonding pressure was applied at room temperature, and the temperature was then raised to the preset diffusion bonding temperature at 50 °C/min. In Stage II, the temperature was held at the diffusion temperature for a designated duration to promote interfacial bonding. In Stage III, after isothermal diffusion was completed, the specimens were cooled to room temperature in the furnace. After welding, the diffusion-bonded joints were sectioned and sampled, as illustrated in Figure 2c. Specimens were then prepared for mechanical property testing and characterization of the metallographic microstructure.

The optimal diffusion bonding temperature for magnesium alloys generally falls within the range of 0.6–0.8 Tm, where Tm is the absolute melting temperature of the alloy [32]. Based on the physical parameters of the MDF Mg-Gd-Y-Zn-Zr rare-earth magnesium alloy matrix employed in this study, the diffusion bonding temperature was determined to be 470 °C. Under this temperature condition, a constant bonding pressure of 4 MPa was applied, and the pressure and temperature were maintained for 30 min to complete vacuum solid-state diffusion bonding of the rare-earth magnesium alloy.

### 2.3. Electroshock Treatment

To further improve the mechanical properties of diffusion-bonded joints of Mg-Gd-Y-Zn-Zr rare-earth magnesium alloy and optimize the interfacial bonding state and microstructure, EST was applied to the bonding interface of the diffusion-bonded joints at various current densities. This approach aims to regulate the microstructure and interfacial bonding characteristics of the joints. The detailed diffusion bonding parameters and the EST parameters used in the experiments are listed in Table 2. The EST experiments were performed using a 50 Hz sinusoidal alternating current. The cross-sectional area of the specimen perpendicular to the current direction was 2 mm^2^. The applied peak current densities were 50 and 70 A/mm^2^ for EST−50 and EST−70, respectively. The EST experimental apparatus mainly consists of a power supply system, clamping electrodes, and a real-time temperature acquisition module. The overall equipment setup is illustrated in Figure 2d. The tensile specimen shown in Figure 2c was cut from the cylindrical diffusion-bonded samples for subsequent EST. Prior to the treatment, the specimen surfaces were polished with abrasive papers to ensure good electrical contact with the electrodes. An infrared thermometer was used to monitor the surface temperature variation among the specimens during the EST process. The detailed procedure of the EST is as follows. First, the surface of the EST specimen was coated with black to reduce reflectivity. The pretreated specimen was then firmly clamped between the positive and negative copper electrodes, and the assembly was connected to the pulse power supply via copper wires. After setting and verifying the pulse process parameters, the power supply was switched on to apply EST to the diffusion-bonded joint.

### 2.4. Mechanical Property Test

Room-temperature tensile tests were conducted using a universal material testing machine (SANS model UTM5105, Shenzhen, China). The maximum loading capacity of the testing system is 100 kN, and the tensile rate was set to 0.5 mm/min. The dimensions and sampling location of the tensile specimens are shown in Figure 2c. Three parallel tensile specimens were tested for each condition. All specimens for the different EST conditions were machined from the same diffusion-bonded assembly to minimize variations arising from the initial bonding process. The tensile strength and elongation at fracture are reported as the mean ± standard deviation (SD, *n* = 3). The engineering strain was determined from the change in specimen gauge length during tensile testing relative to the initial gauge length, and the engineering strain at fracture was used as the elongation at fracture to characterize the ductility of the joint. After tensile fracture, the fracture surfaces of the diffusion-bonded joints were examined using scanning electron microscopy (SEM).

### 2.5. Microstructure Characterization Methods

Scanning electron microscopy (SEM), energy-dispersive spectroscopy (EDS), electron backscatter diffraction (EBSD), and X-ray diffraction (XRD) were employed to systematically characterize the microstructure and phase composition of the diffusion-bonded joints of Mg-Gd-Y-Zn-Zr rare-earth magnesium alloy. Metallographic specimens were sequentially ground using abrasive papers from 600# to 3000#, followed by fine polishing using diamond polishing paste with 0.25 μm particle size. After polishing, the interfacial microstructure of the diffusion-bonded joints was observed by SEM. XRD measurements were conducted using an X-ray diffractometer (Bruker model D8 ADVANCE, Karlsruhe, Germany) with Cu Kα radiation. The 2θ scanning range was set from 20° to 80°, with a scanning rate of 1°/min. EBSD characterization was performed using a microscopic system (Guoyi Quantum model 4000Pro, Hefei, China) equipped with an equipped with an EBSD detector (Oxford Instruments model Swift, Abingdon, UK). Prior to testing, the EBSD specimens were mechanically polished, ultrasonically cleaned in acetone, and then electrolytically polished in an ethanol electrolyte containing 10% perchloric acid. The EBSD scanning step size was set to 1 μm to collect raw crystallographic data. The EBSD data were analyzed using AZtecCrystal 2.1 software (Oxford Instruments, Abingdon, UK). KAM was calculated using a 3 × 3 square kernel with the “perimeter only” option enabled and a maximum misorientation threshold of 5°. Misorientations greater than 5° were excluded from the KAM calculation. The GND density was estimated from local crystallographic orientation gradients using a 5 × 5 square kernel and a maximum misorientation threshold of 5°. For the Mg matrix, a dominant a-type Burgers vector of 1/3<112(-)0>, with a magnitude of 0.321 nm, and a geometrical factor of (*α* = 3) were adopted. One representative EBSD region was analyzed for each condition, containing 4693, 204, and 267 grains for MDF, EST−0, and EST−50, respectively. For the interfacial spatial analysis, a ±50 μm region centered on the bonding interface was selected, and the coefficient of variation of GND density was calculated as *CV* = *SD*/mean × 100%. The {0001} pole figures of the Mg phase were calculated using an equal-area projection on the XY plane and the upper hemisphere, with a half-width of 10°, sample symmetry enabled and the acquisition surface (CS1) as the reference coordinate system.

## 3. Results and Discussion

### 3.1. Surface Temperature Variation in EST

**Figure 3:**
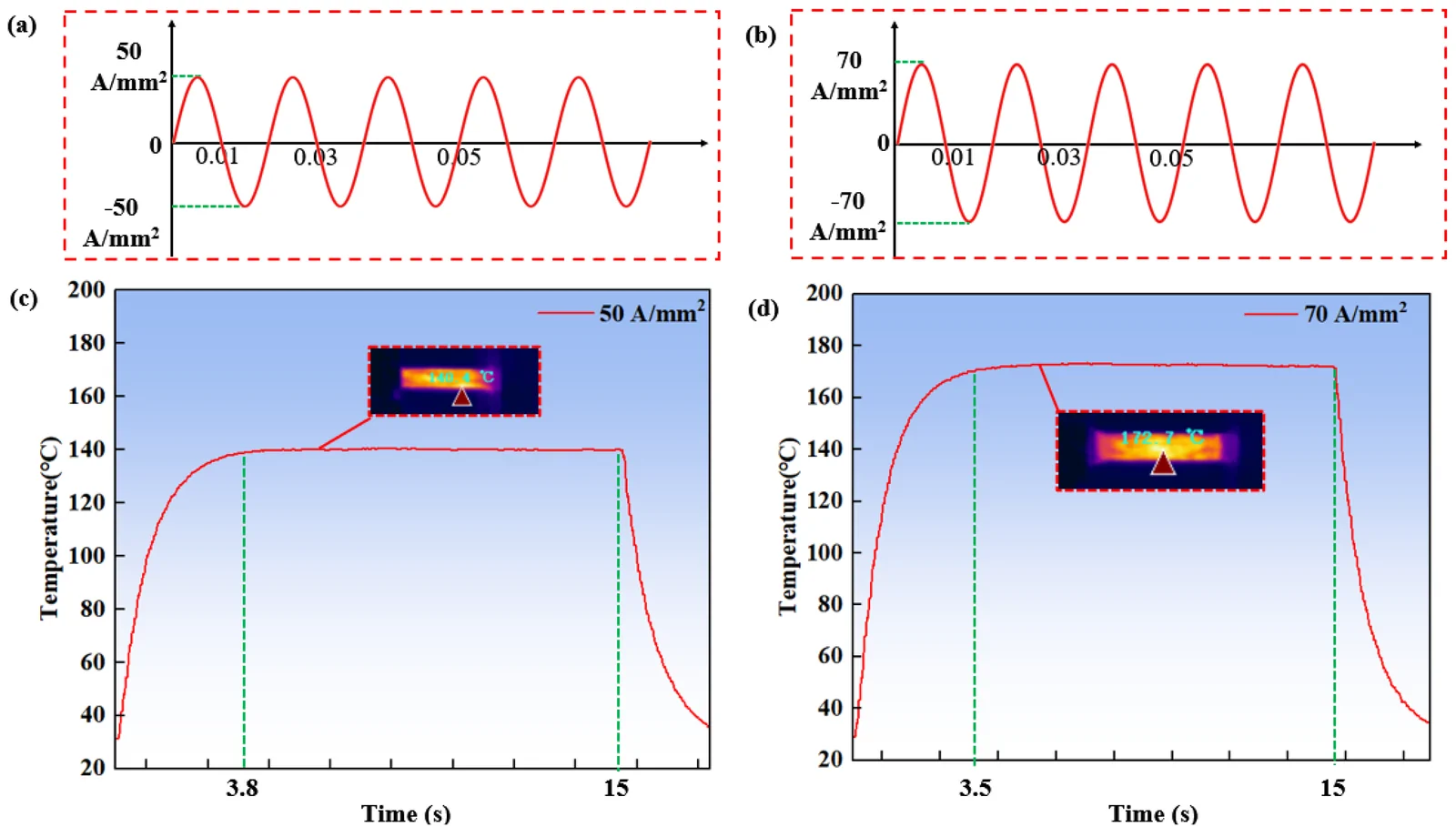
Schematic current waveforms and surface temperature evolution of the specimen during EST: (**a**,**c**) EST−50; (**b**,**d**) EST−70.

Figure 3 presents the schematic sinusoidal AC waveforms together with the surface temperature evolution of the specimens during EST at different current densities. For a given constant current density, the surface temperature rises rapidly immediately after the current is applied. Under current densities of 50 A/mm^2^ and 70 A/mm^2^, the peak temperatures are reached within 3.5 s and 3.8 s, respectively. As the treatment progresses, Joule heat generation and ambient heat dissipation gradually reach a dynamic equilibrium, and the surface temperature eventually stabilizes. At a current density of 50 A/mm^2^, the steady-state maximum temperature of the specimen reached 140.4 °C. When the current density was increased to 70 A/mm^2^, the Joule heating effect was significantly enhanced, and the maximum temperature at the equilibrium stage rose to 172.7 °C. Overall, as the current density increases, the Joule heating efficiency improves remarkably, the temperature rise rate of the specimen accelerates, and the steady-state surface temperature increases correspondingly. It should be noted that no separate thermal-control treatment was performed at temperatures comparable to those reached during EST for the same treatment duration of 15 s. Therefore, the individual contributions of Joule heating and possible athermal effects associated with the applied electric current cannot be quantitatively distinguished in the present study.

### 3.2. Mechanical Property

**Figure 4:**
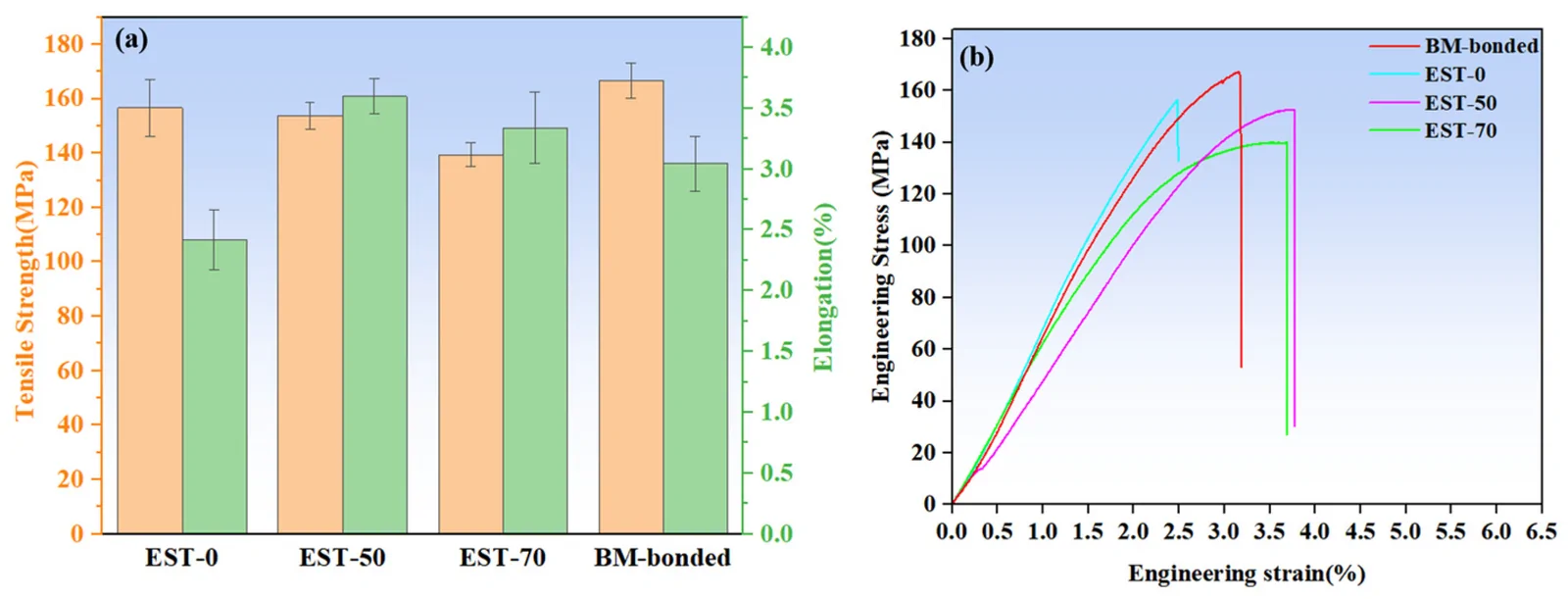
Mechanical properties of diffusion-bonded joints under different EST conditions at 470 °C: (**a**) tensile strength and elongation at fracture, presented as mean ± standard deviation (SD, *n* = 3), with error bars representing ± SD; (**b**) representative engineering stress–strain curves.

Tensile strength and elongation are important indicators to verify the improvement effect of EST on the mechanical properties and fracture behavior of Mg-Gd-Y-Zn-Zr rare-earth magnesium alloy diffusion-bonded joints. The tensile property results of the diffusion-bonded Mg-Gd-Y-Zn-Zr rare-earth magnesium alloy joints under different EST conditions are presented in Figure 4. The as-received MDF alloy exhibits a tensile strength of approximately 285 MPa. After exposure to the diffusion bonding cycle at 470 °C, 4 MPa, and 30 min, the tensile strength of the base metal decreases to approximately 166.67 ± 6.38 MPa. This post-bonding base-metal state, denoted as “BM-bonded” in Figure 4, is used as the reference for evaluating the mechanical properties of the diffusion-bonded joints. For the EST−0, EST−50, and EST−70 conditions, tensile fracture consistently occurred at the diffusion bonding interface. Compared with the original base material, the high-temperature environment during diffusion bonding reduces both the tensile strength and elongation of the multi-directionally forged magnesium alloy. As shown in Figure 4a, the tensile strength of the diffusion-bonded joint fabricated at 470 °C is 156.62 ± 10.28 MPa, and its elongation decreases from 3.05 ± 0.22% to 2.42 ± 0.25%. This tensile strength corresponds to approximately 94% of that of the base material after the diffusion bonding thermal cycle. Previous studies on diffusion-bonded Mg-RE alloys reported strong dependence of joint performance on bonding conditions, a tensile strength of 279 MPa with 2.8% elongation for heat-treated Mg-6Gd-3Y joints [22], and a shear strength of 152 MPa (98.7% of the base material) for Mg-Gd-Y-Zn-Zr alloy bonded with a Cu interlayer [33]. For Mg/Al dissimilar diffusion-bonded joints, a tensile strength of approximately 80.3 MPa has been reported after subsequent annealing at 250 °C [34]. These results provide useful context for the present joint, although direct comparison is limited by differences in material state, bonding process, post-treatment, and test mode. After EST at a current density of 70 A/mm^2^, the tensile strength of the diffusion-bonded joint reaches 139.4 MPa, while its elongation increases from 2.42 ± 0.25% to 3.34 ± 0.15%. The tensile strength retains 83% of that of the diffusion-bonded base metal. At a current density of 50 A/mm^2^, the tensile strength and elongation of the diffusion-bonded joint are 153.79 ± 4.85 MPa and 3.60 ± 0.15%, respectively. Among the investigated EST conditions, 50 A/mm^2^ provides the most favorable combination of tensile strength and ductility. The tensile strength remains close to that of the untreated diffusion-bonded joint, while the elongation at fracture increases by approximately 48.8%, from 2.42 ± 0.25% to 3.60 ± 0.15%. When the current density is further increased to 70 A/mm^2^, the higher thermal input may promote more pronounced dislocation recovery and annihilation, thereby reducing the contribution of dislocation strengthening and lowering the tensile strength. These results indicate that an appropriate EST current density can improve the ductility of the diffusion-bonded joint while largely maintaining its tensile strength. EST regulates the microstructure of the diffusion bonding interface and matrix, providing a potential approach for improving the mechanical performance of rare-earth magnesium alloy diffusion-bonded joints.

### 3.3. Tensile Fracture Morphology

**Figure 5:**
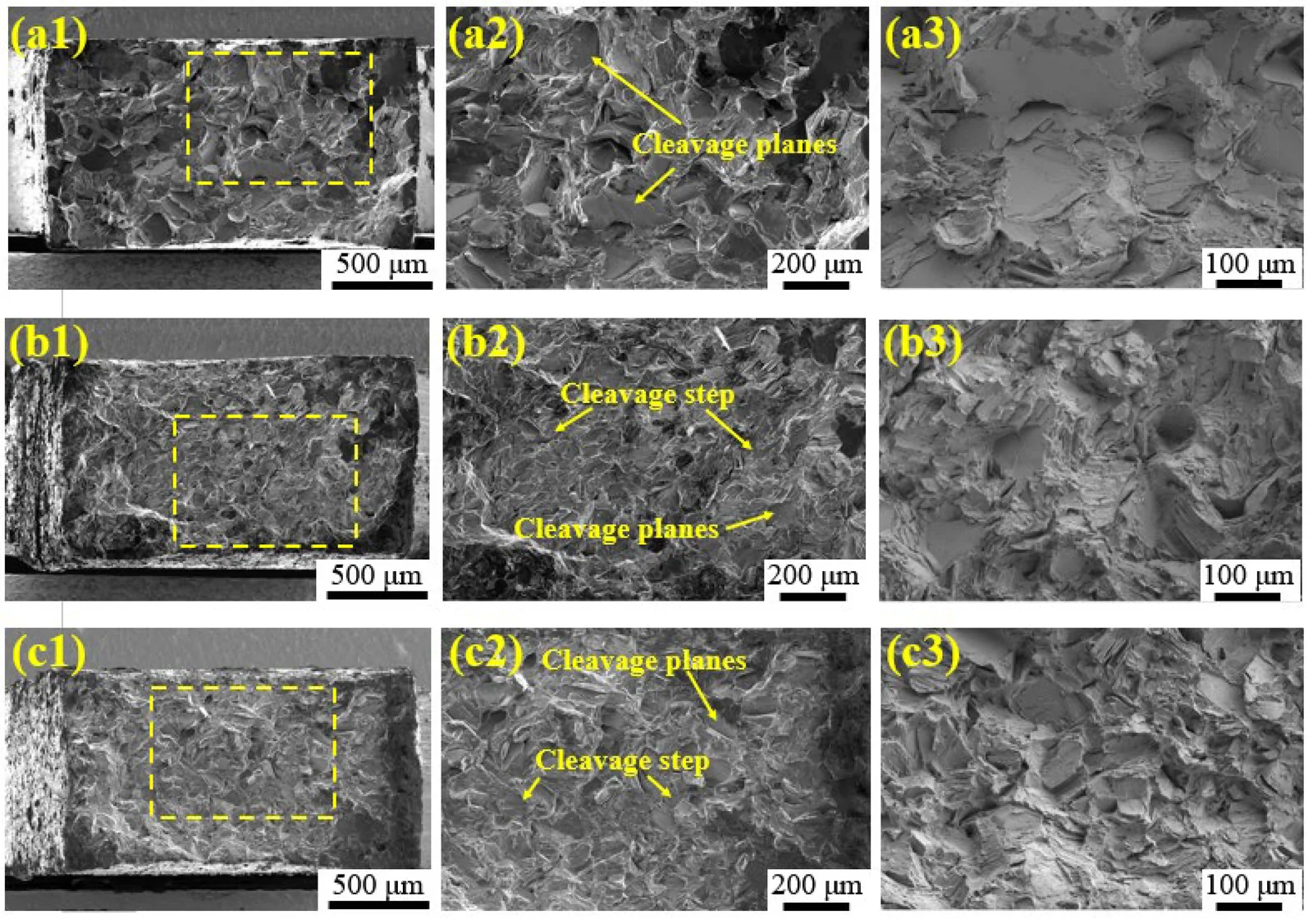
Tensile fracture morphologies of Mg-Gd-Y-Zn-Zr rare-earth magnesium alloy under different states: (**a1**–**a3**) EST−0, (**b1**–**b3**) EST−50, (**c1**–**c3**) EST−70. The yellow dashed boxes in (**a1**), (**b1**), and (**c1**) indicate the regions corresponding to the higher-magnification images in (**a2**), (**b2**), and (**c2**), respectively.

Figure 5 presents the tensile fracture morphologies of Mg-Gd-Y-Zn rare-earth magnesium alloy diffusion-bonded joints under different treatment conditions. The fracture surfaces of all diffusion-bonded joints consist of cleavage planes and abundant tearing ridges, and the fracture mode is identified as transgranular quasi-cleavage fracture. The as-bonded diffusion joint shown in Figure 5(a1–a3) exhibits numerous large cleavage planes on the fracture surface, reflecting the poor plasticity of the untreated joint. After EST, most of the cleavage planes disappear, indicating that the diffusion-bonded joint fractures predominantly in a quasi-cleavage mode, as shown in Figure 5(c1–c3). For the diffusion-bonded joint treated at 50 A/mm^2^, the size and number of cleavage planes decrease significantly, as shown in Figure 5(b1–b3).

### 3.4. Microstructure Characterization

#### 3.4.1. SEM Characterization of Diffusion Interface Under Different States

**Figure 6:**
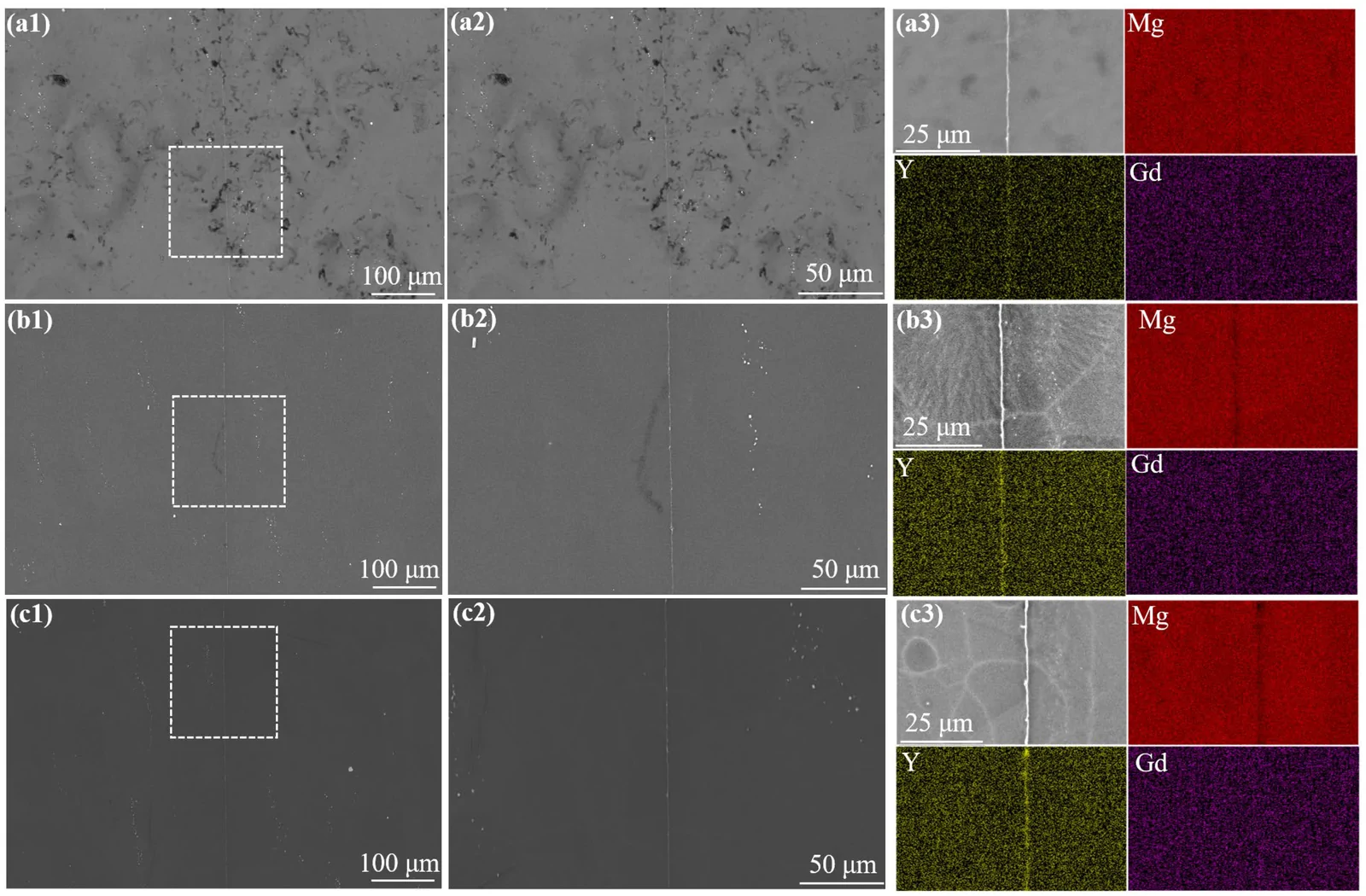
SEM images and EDS mappings of diffusion bonding interfaces under different electroshock parameters: (**a1**–**a3**) EST−0, (**b1**–**b3**) EST−50, (**c1**–**c3**) EST−70. The white dashed boxes in (**a1**), (**b1**), and (**c1**) indicate the regions corresponding to the higher-magnification images in (**a2**), (**b2**), and (**c2**), respectively.

The bonding interfaces of both the as-bonded and electroshock-treated diffusion-bonded joints were characterized using scanning electron microscopy in backscattered electron (SEM-BSE) mode. Figure 6(a1–c1) present the SEM images of the bonding interfaces under different processing parameters. As shown in the figure, the diffusion interface of the diffusion-bonded joint fabricated at 470 °C is well bonded, without obvious welding defects such as voids. EDS analysis was performed on the diffusion bonding interface, as shown in Figure 6(a3–c3). The elemental distributions reveal local enrichment of Y in the intermediate bonding region, with a higher Y signal at the diffusion interface than in the adjacent base metal. Figure 6(b3,c3) show the elemental distribution characteristics at the interface of diffusion-bonded joints after EST−50 and EST−70, respectively. The primary reason is that plenty of micro-voids inherently exist at the bonding interface in the early diffusion-bonding stage. Local high-temperature zones occur at the contact asperities of the diffusion interface under coupled thermo-electro-mechanical loading. These micro-asperities undergo plastic deformation, accompanied by interdiffusion of atoms across the interface and the precipitation of newly formed secondary phases, which jointly promote the gradual healing and closure of the micropores [35]. During EST, the diffusion-bonded interface possesses high intrinsic defect density and considerable electrical resistance contrast. The SEM observations in the present study indicate that a well-bonded interface was obtained after diffusion bonding, and no obvious interfacial voids were observed after EST. In addition, a reduced presence of bright second-phase particles is observed after diffusion bonding compared with the MDF material.

#### 3.4.2. EBSD Characterization of Diffusion Interface Under Different Parameters

**Figure 7:**
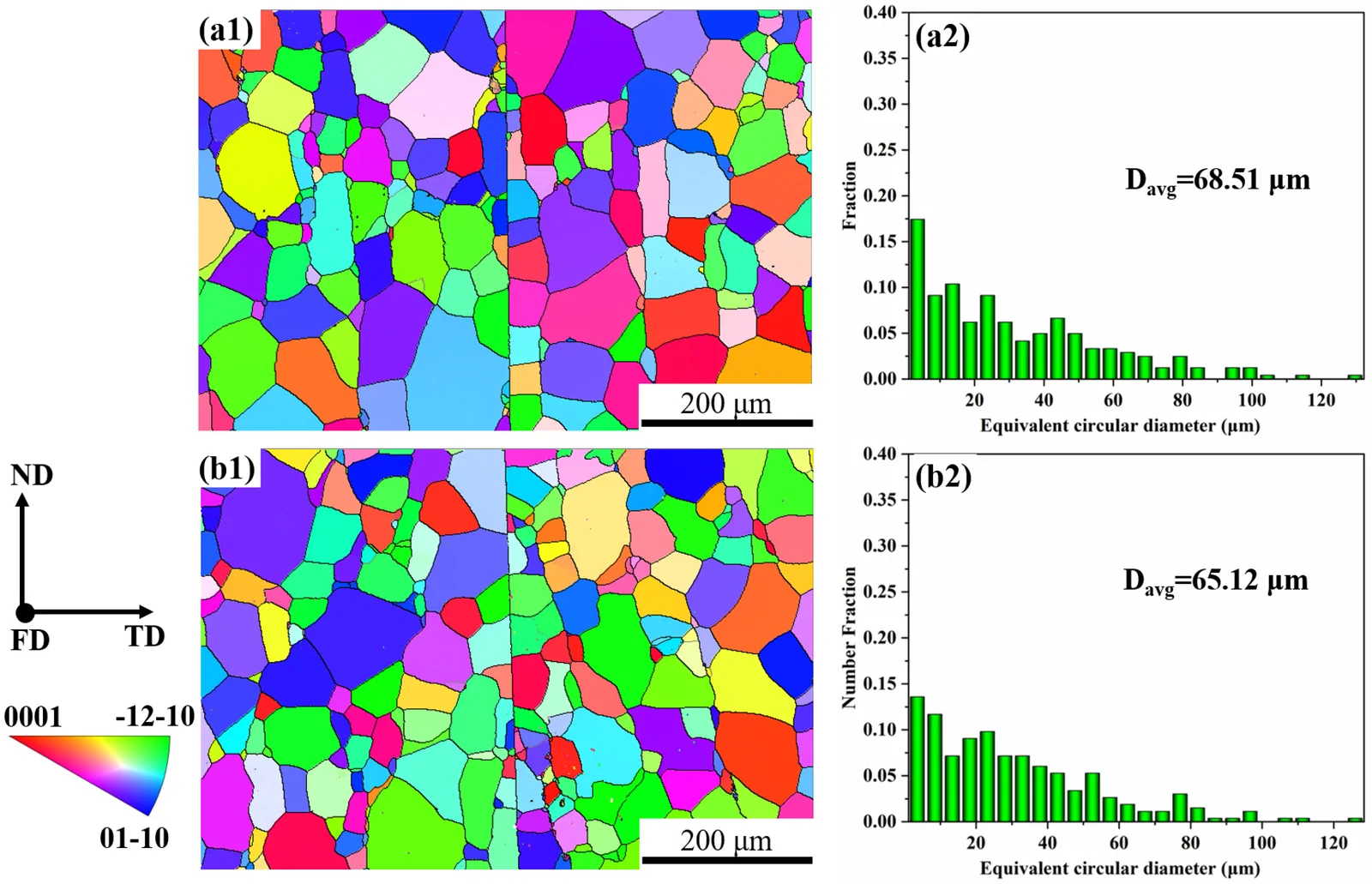
IPF maps and grain size statistics at the interface of diffusion bonding joints under different treatment conditions: (**a1**,**a2**) EST−0, (**b1**,**b2**) EST−50.

To further elucidate the microscopic mechanism underlying the improvement in elongation after fracture of diffusion-bonded joints by EST, EBSD characterization was performed on the welding interface under the EST−50 condition. The evolution of the internal microstructure of the joint before and after EST was analyzed. Figure 7(a1,a2) present the EBSD characterization results of the diffusion-bonded joint fabricated at 470 °C. During diffusion bonding at 4 MPa and 470 °C, the applied pressure induces localized microplastic deformation of the microscopic asperities on the faying surfaces, thereby introducing high stored energy in the form of dislocations within a thin interfacial layer. Upon subsequent high-temperature holding, recovery and static recrystallization preferentially occur in this high-dislocation-density interfacial region, producing fine equiaxed recrystallized grains through subgrain coalescence and the gradual transformation of subgrain boundaries into high-angle grain boundaries. Furthermore, the welding temperature exceeds the recrystallization temperature of the Mg-Gd-Y-Zn-Zr rare-earth magnesium alloy, thereby providing favorable thermodynamic conditions for recrystallization in the diffusion-bonded joints [36]. The high-temperature environment during welding significantly enhances grain boundary mobility, leading to abnormal grain growth and coarsening. Consequently, significant grain growth is observed throughout the bonded specimen. Compared with the original multi-directionally forged base material, the average grain size of the rare-earth magnesium alloy increases from an initial 14.92 μm to over 68.51 μm after diffusion bonding. During diffusion bonding, grain coarsening is dominated by atomic diffusion in the magnesium matrix. Under the combined effect of these two factors, the tensile strength of the diffusion-bonded joint prepared at 470 °C is lower than that of the original forged base metal. Comparative SEM observations further reveal a qualitatively reduced presence of bright second-phase particles after diffusion bonding. These combined microstructural changes account for a substantial part of the decrease in tensile strength from approximately 285 MPa in the MDF material to approximately 156 MPa in the diffusion-bonded joint. After EST, the average grain size of the diffusion-welded joint was 65.12 μm, showing no obvious change.

**Figure 8:**
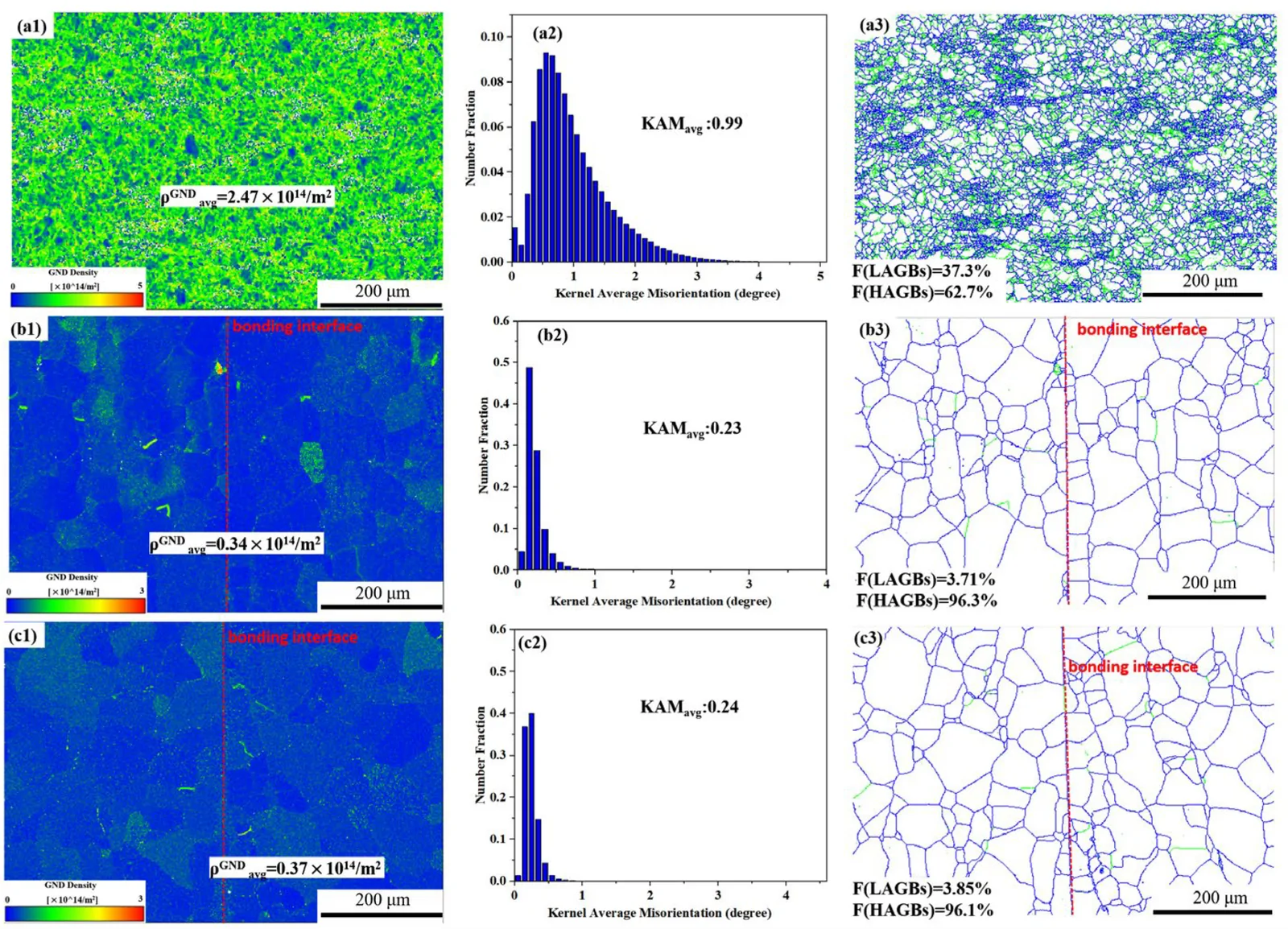
GND density, KAM, and grain-boundary characteristics of the rare-earth magnesium alloy under different conditions: (**a1**–**a3**) MDF Magnesium alloy, (**b1**–**b3**) EST−0, (**c1**–**c3**) EST−50. The green and blue lines in (**a3**), (**b3**), and (**c3**) represent low-angle grain boundaries (LAGBs) and high-angle grain boundaries (HAGBs), respectively.

Figure 8 presents the geometrically necessary dislocation (GND), kernel average misorientation (KAM), and grain boundary distributions of the multi-directionally forged Mg-Gd-Y-Zn-Zr rare-earth magnesium alloy base material, the diffusion bonding base metal, and the diffusion-bonded joint. Figure 8(a1–c1) compare the evolution of geometrically necessary dislocation (GND) density in the MDF magnesium alloy, diffusion-bonded joint, and diffusion joint after EST. Figure 8(a1–c1) compare the GND density distributions of the MDF alloy, EST−0 joint, and EST−50 joint. The MDF material exhibits a relatively high GND density, with an average value of 2.47 × 10^14^ m^−2^. After diffusion bonding, the average GND density decreases markedly to 0.34 × 10^14^ m^−2^, consistent with substantial recovery and recrystallization during high-temperature holding. After EST−50, the average GND density remains comparable at 0.37 × 10^14^ m^−2^, indicating that EST−50 does not appreciably alter the overall GND density. A quantitative analysis of the ±50 μm interfacial region further shows that the coefficient of variation of the GND density decreases from 80.46% for EST−0 to 57.68% for EST−50. Although the mean GND density does not decrease, the lower CV suggests reduced relative spatial heterogeneity of the GND distribution within the analyzed interfacial region.

The average KAM value decreases from 0.99° in the MDF condition to 0.23° after diffusion bonding and remains nearly unchanged at 0.24° after EST−50. Thus, EST−50 does not appreciably alter the overall level of local lattice misorientation. The reduced spatial variability of GND density near the interface, rather than a decrease in its average value, suggests a possible redistribution of local orientation gradients after EST. This change may contribute to more coordinated local deformation and is consistent with the increased elongation at fracture. Figure 8(a3–b3) show the orientation distribution characteristics of low-angle and high-angle grain boundaries for the original base material, the EST−0 specimen, and the EST−50 specimen. Grain boundaries with a misorientation angle of less than 15° between adjacent grains are defined as low-angle grain boundaries (LAGBs, marked by green lines), whereas those with a misorientation angle greater than 15° are defined as high-angle grain boundaries (HAGBs, marked by blue lines). As shown in Figure 8(a3–c3), the LAGB/HAGB fractions change from 37.3%/62.7% in the MDF condition to 3.71%/96.3% after diffusion bonding, reflecting pronounced microstructural recovery and recrystallization. After EST−50, the corresponding fractions are 3.85%/96.1%, indicating that EST−50 causes little change in the overall grain-boundary misorientation distribution within the analyzed region.

**Figure 9:**
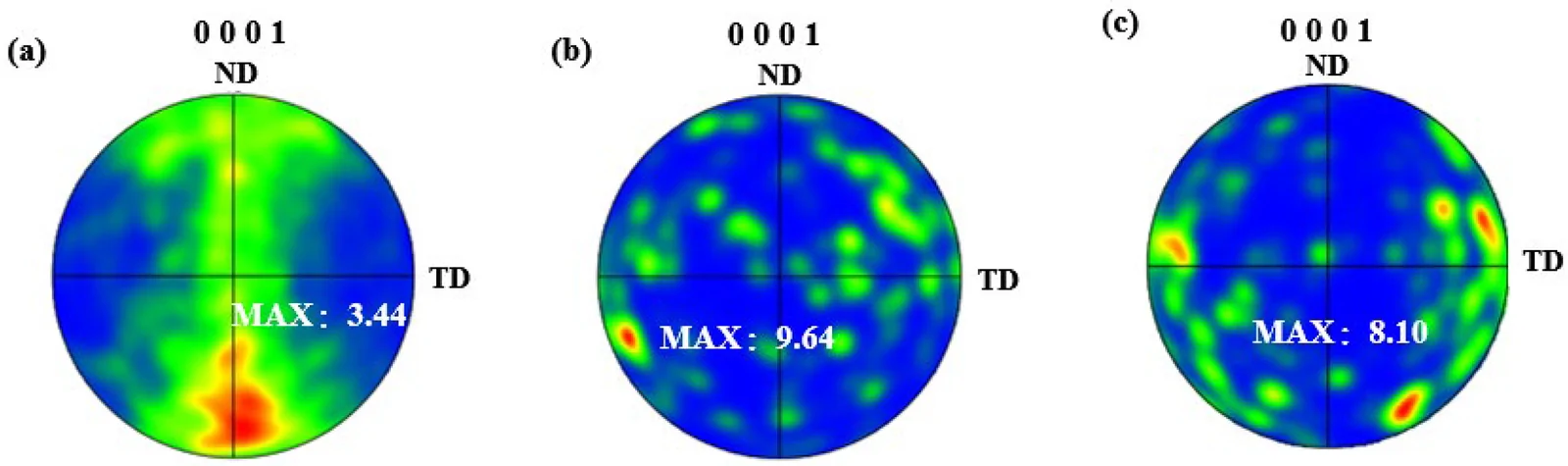
{0001} pole figures of samples under different states: (**a**) MDF alloy, (**b**) EST−0, (**c**) EST−50.

The {0001} pole figures obtained under different processing parameters are presented in Figure 9a–c. Owing to the multidirectional loading path during multi-directional forging (MDF), the base material undergoes grain fragmentation and rotation in multiple spatial directions, which effectively breaks the preferential alignment of a single orientation and suppresses the formation of a strong basal texture. Consequently, its initial texture intensity is only 3.44. During diffusion bonding, grain coarsening occurs, and the residual strain is gradually released under high-temperature holding. Meanwhile, crystallographic orientation evolution occurs during the high-temperature bonding cycle. The reduction in the number of fine, relatively randomly oriented grains and the preferential growth of grains with favorable orientations may contribute to the enhanced basal texture. Accordingly, the maximum texture intensity increases from 3.44 to 9.64 after diffusion bonding. In summary, the combined effect of high-temperature grain coarsening and preferential orientation evolution during diffusion bonding ultimately leads to a simultaneous increase in both grain size and texture intensity. For the specimens subjected to EST, the maximum texture intensity within the analyzed EBSD region decreases from 9.64 to 8.10, suggesting a tendency toward texture weakening. The observed decrease in texture intensity may be associated with the combined influence of Joule heating and possible athermal effects of the applied electric current, which may promote dislocation motion and crystallographic orientation adjustment [37].

### 3.5. Mechanisms of Strength–Ductility Synergy Induced by EST

**Figure 10:**
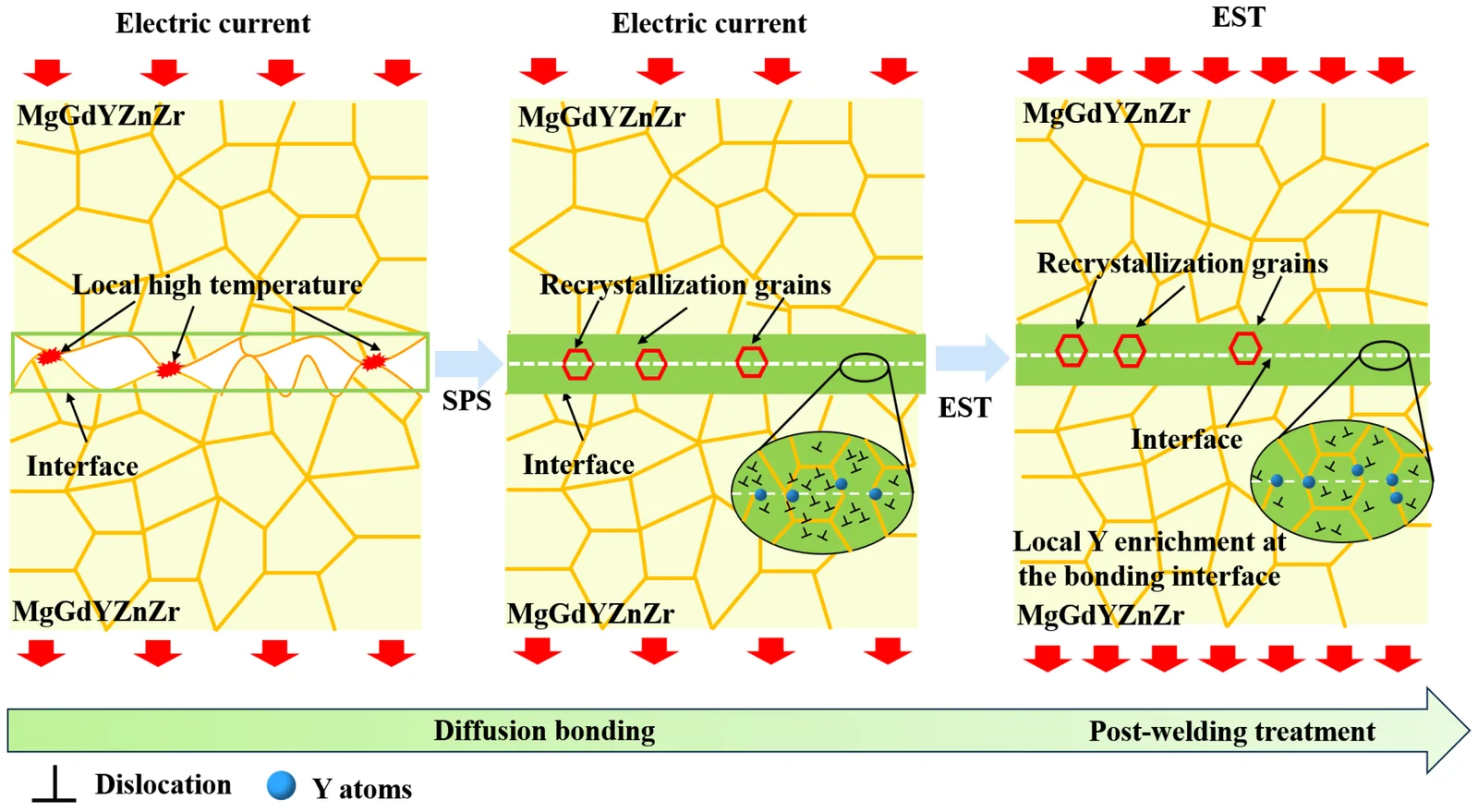
Interfacial strengthening mechanism of rare-earth magnesium alloy diffusion bonding joint under EST. The dashed lines indicate the original bonding interface, and the orange lines represent grain boundaries.

Figure 10 illustrates the mechanism of plasticity improvement in diffusion-bonded joints of Mg-Gd-Y-Zn-Zr rare-earth magnesium alloy subjected to EST. In the initial stage of diffusion bonding, limited by the process temperature and surface roughness of the specimens, only local point contact is established at the bonding interface. Under the action of the pulsed current, local high temperatures are generated at the interfacial contact regions, inducing significant plastic deformation at the interface and effectively improving the metallurgical bonding ratio of the diffusion-bonded interface. The improvement in the interfacial bonding ratio further broadens the atomic diffusion channels. Meanwhile, EST effectively promotes dislocation motion at the interface and results in a more uniform spatial distribution of dislocations. Under the synergistic effects of these mechanisms, the ductility of the Mg-Gd-Y-Zn-Zr rare-earth magnesium alloy diffusion-bonded joints is enhanced, as evidenced by the increased elongation at fracture, while only a limited change in the ultimate tensile strength is observed.

## 4. Conclusions

(1) Under the conditions of a bonding temperature of 470 °C, a holding time of 30 min, and an axial pressure of 4 MPa, the tensile strength of the diffusion-bonded joint reached 156.62 MPa, corresponding to approximately 94% of that of the base metal. Different EST current densities were subsequently applied to the as-bonded joints. Among the investigated EST conditions, 50 A/mm^2^ provided the most favorable combination of tensile strength and ductility. After EST at 50 A/mm^2^, the tensile strength of the joint remained at approximately 153.79 MPa. Meanwhile, the elongation after fracture increased by 48.8% compared with the untreated sample, reaching 3.60%.
(2) Microstructural characterization revealed that the diffusion interfaces exhibited excellent metallurgical bonding before and after EST, without detectable pores or interfacial bonding defects. The diffusion bonding process induced pronounced static recrystallization at the interface, leading to grain coarsening of the multidirectional forged matrix alloy, with the average grain size increasing from 14.9 μm to 68.5 μm. EDS elemental analysis revealed local enrichment of Y at the diffusion bonding interface.
(3) The average GND density and KAM value remain nearly unchanged after EST-50. However, within the ±50 μm interfacial region, the coefficient of variation of the GND density decreases from 80.46% for EST−0 to 57.68% for EST−50, suggesting reduced relative spatial heterogeneity of the GND distribution. Meanwhile, the maximum texture intensity within the analyzed EBSD region decreases from 9.64 to 8.10, indicating a tendency toward texture weakening. These microstructural changes may be associated with dislocation rearrangement and crystallographic orientation adjustment under the combined influence of Joule heating and possible athermal effects of the applied current, and may contribute to the enhanced ductility of the joint.

## Figures and Tables

**Table 1:** Chemical composition of Mg-Gd-Y-Zn-Zr magnesium alloy (wt.%).

Elements	Gd	Y	Zr	Zn	Fe	Ni	Si	Mg
Weight fraction	8.17	2.82	0.30	0.03	<0.01	<0.01	<0.05	Bal

**Table 2:** Diffusion bonding experimental parameters.

Sample	Temperature (°C)	Pressure (MPa)	Time (min)	Current Density (A/mm^2^)	Processing Times (s)	Frequency (Hz)
EST−0	470	4	30	-	-	-
EST−50				50	15	50
EST−70				70	15	50

## Data Availability

The original contributions presented in this study are included in the article. Further inquiries can be directed to the corresponding author.
